# Factors associated with the use of combined nutritional complementary and alternative medicine among southern US older adults: results from the study of aging II

**DOI:** 10.3389/fnut.2025.1595919

**Published:** 2025-08-11

**Authors:** Kristen Allen-Watts, Deanna Rumble, Taylor Taylor, Andrew Sims, Lisa H. Antoine, Georgiana Logan, Cynthia J. Brown, Thomas W. Buford, Burel R. Goodin, Andrea Cherrington, Richard Kennedy

**Affiliations:** ^1^Department of Medicine, University of Alabama at Birmingham, Birmingham, AL, United States; ^2^Department of Psychology and Counseling, University of Central Arkansas, Conway, AR, United States; ^3^Jackson Heart Study, University of Mississippi Medical Center, Jackson, MS, United States; ^4^United States Patent and Trademark Office, Alexandria, VA, United States; ^5^Department of Health Science, Marshall University, Huntington, WV, United States; ^6^Department of Medicine, Louisiana State University Health Sciences Center, New Orleans, LA, United States; ^7^Department of Anesthesiology, Washington University School of Medicine in St. Louis, St. Louis, MO, United States

**Keywords:** older adults, dietary supplements, vitamins, minerals, complementary and alternative medicine

## Abstract

**Background:**

Older adults use nutritional complementary and alternative medicine (CAM) to reduce the risk of (or treat) non-communicable conditions and diet deficiencies. While prior research has explored the individual use of dietary supplements, vitamins, and minerals among older adults, few studies have examined factors influencing the combined use of these modalities, especially among Southern, older adults in the United States.

**Methods:**

Data were extracted from 419 participants from the University of Alabama at Birmingham Study of Aging II, a population-based longitudinal study of mobility among community-dwelling older adults. Self-reported data, including insurance and rural residence status, was collected. Comorbidity burden was assessed using the Charlson Comorbidity Index. Participants reported their use of non-prescribed medications, including dietary supplements, vitamins, and minerals. Logistic regression was used to identify factors associated with the use of combined nutritional CAM modalities.

**Results:**

We found a statistically significant association between sex (*p* < 0.001), age (*p* = 0.024), rural living status (*p* = 0.008), and education (*p* < 0.001) in use of combined nutritional CAM (dietary supplements, vitamins, and minerals). For the use of vitamins and minerals only, our findings suggest a significant association between sex (*p* = 0.027), age (*p* < 0.001), and education (*p* = 0.009). Lastly, for the use of dietary supplements only, our findings suggest a significant association between age (*p* = 0.050) and education (*p* = 0.002).

**Conclusion:**

Our study addresses a critical gap by examining the sociodemographic and chronic disease burden predictors of concurrent use of combined nutritional CAM modalities among older adults in the Southern United States. Such insights can help inform public health strategies and clinical guidance aimed at supporting the health and well-being of older adults, particularly as they navigate complex health decisions in the context of aging.

## 1 Introduction

As the adult population continues to age, the prevalence of non-communicable diseases, nutrient deficiencies, and age-related conditions also increases; posing significant risks to the overall health, well-being, and quality of life of older adults (1–3). In response to these challenges, a substantial proportion of older adults use Complementary and Alternative Medicine (CAM) (4), a broad category of medical and health-related products and practices that fall outside the scope of conventional Western medicine (5).

The National Center for Complementary and Integrative Health (NCCIH) categorizes CAM into three primary domains: psychological (e.g., mindfulness, spiritual practices, psychotherapy), physical (e.g., massage, spinal manipulation), and nutritional (e.g., special diets, herbs, food as medicine, vitamins, and minerals) (5). Among these domains, nutritional CAM is particularly prevalent among older adults (6, 7), with dietary supplements being one of the most commonly used modalities (8).

Dietary supplements encompass a broad category of ingestible products designed to complement the diet and support specific health outcomes. These products include a wide range of ingredients such as vitamins, minerals, botanicals, herbs, compounds, live microbials, and amino acids (9–11). Within this broad category, vitamins and minerals represent a distinct and essential subset. Vitamins are classified based on their solubility. Fat-soluble vitamins (A, D, E, and K) are generally excreted if not absorbed (12, 13). Minerals, such as calcium, sodium, potassium, copper, iodine, and zinc, are inorganic elements that also play a critical role in maintaining health and must be consumed through dietary sources (14). While all vitamins and minerals are considered dietary supplements when taken in supplement form, not all dietary supplements are limited to vitamins and minerals (10, 11, 15). This distinction is important, as it highlights the diverse nature of dietary supplements and the varied role it plays in supporting health and nutritional needs, particularly among populations with limited dietary intake, chronic health conditions, or increased nutrient requirements.

While extensive research has explored patterns of nutritional CAM utilization among older adults (7, 16), little is known about the combined use of nutritional CAM modalities in this population. This gap is particularly relevant in the Southern United States, where residents face unique health disparities (17). Compared to other regions, the South has lower rates of urbanization and higher rates of chronic disease and premature mortality, all of which contribute to reduced access to conventional healthcare services and further complicate the nutritional landscape (18). Furthermore, the South’s distinct dietary patterns, often characterized by high consumption of fried and processed foods, have been linked to increased risk of cardiometabolic diseases (19, 20) and contribute to micronutrient deficiencies (21, 22), such as vitamin D, magnesium, and calcium, making nutritional CAM usage more common or necessary in this population. The use of multiple nutritional CAM can pose significant health risks, including harmful interactions with medications, excessive nutrient intake, and compounded side effects, particularly in older adults. These risks are further exacerbated by inconsistent regulation and labeling of supplement products, which may lead to contamination or inaccurate dosing (23). Given these concerns, the present study used data from the Study of Aging (SOA) II to examine sociodemographic and disease burden factors associated with the combined use of nutritional CAM modalities. Specifically, we explored patterns in the use of (1) combined nutritional CAM, (2) vitamins and minerals only, and (3) dietary supplements only among older adults in the South.

## 2 Methods

### 2.1 Data source and sample

Data were extracted from the University of Alabama at Birmingham (UAB) SOA II (NIA # R01-AG15062), a population-based, longitudinal study that examined the frequency and magnitude of community mobility associated with specific events (e.g., hospitalization and emergency room visits) and reasons for racial disparities in community mobility among community-dwelling older adults (24). Participants were recruited from two prior studies: the UAB SOA I (1999–2008), a prospective, observational study of a population-based sample of 1,000 community-dwelling Medicare beneficiaries, and the State of Alabama Charting the Course, a Long-Term Needs Assessment (25). Telephone visits and dietary recalls were completed monthly for 36-months. The inclusion criteria for SOA II were individuals who self-identified as African American or White, 75 years of age or older, and completed measures regarding sociodemographics and medication history from June 2010 to August 2011. Participant retention and response rates in the UAB SOA II were high, with exceptions being attrition due to death or relocation (26). The current study is a secondary analysis of the larger study. Measures and procedures for the current study included sociodemographic factors and participants who answered questions related to their medication history. The protocol was reviewed and approved by the Institutional Review Board at UAB (IRB-090803002).

### 2.2 Measures

Self-reported sociodemographic data were collected, including age, sex (male or female), race (African American or White), and education (less than high school (HS), completed HS, some college, college graduate, some graduate/professional school, and graduate/professional degree). Insurance status was also recorded (Medicare, Medicaid, private, Veteran’s Administration (VA) coverage). Additionally, rural residence status was determined using the “Am I Rural” tool, which classifies locations as rural based on federal program definitions (27).

To assess overall health burden, the comorbidity score was calculated using the Charlson comorbidity index (CCI), a validated measure that assigns weighted values to a range of chronic conditions to estimate 10-year mortality risk (28) Comorbidities were considered verified if the participant reported the condition and was taking medication for it, if it was documented in a physician-completed questionnaire, or if it appeared in a hospital discharge summary. The total comorbidity score was derived from the number and severity of conditions listed in the CCI.

### 2.3 The categorization and definitions of nutritional CAM (dietary supplements & vitamins and minerals)

Participants were asked to provide a comprehensive list of all medications and health-related products they were currently using. This included prescription and over-the-counter medications (including injectables), as well as dietary supplements, vitamins, aspirin, sinus medicines, herbal preparations, bowel medications, sleep aids, mouth rinses, and other health-related products. This information was collected during the initial in-home visit, with updates recorded at each follow-up visit based on any reported changes to the participants regimen.

In accordance with common definitions in the literature, this study classified dietary supplements to include a wide range of herbs and botanicals, such as glucosamine chondroitin, echinacea (11, 29). Responses were categorized as vitamins based on standard classifications, including solubility (water-soluble vs. fat-soluble) and biological function. These included vitamins A, B-complex, C, D, E, and K (30). Minerals were categorized according to established classifications as either major minerals, such as calcium, sodium, and potassium, or trace minerals, such as iron (14). For the purposes of this study, the use of dietary supplements, vitamins and minerals was classified as nutritional CAM if participants reported using these products at any point during the 36-month data collection period.

### 2.4 Statistical analysis

Data were analyzed using SPSS version 28.0. Descriptive statistics were computed for the sample, and all data were presented as percentages or as means and standard deviations (SD). To examine the factors influencing the use of different types of combined nutritional CAM, the dependent variables included in the analyses were the use of combined dietary supplements, vitamins, and minerals (group 1), the use of vitamins and minerals only (group 2), and the use of dietary supplements only (group 3). Each dependent variable was dichotomized as yes/no. Insurance status was a multiple response question (Medicaid, Medicare, VA, or private), therefore, we re-categorized to include all possible options. Highest education was re-categorized to include less than High School, High School, Some College, College graduate, some graduate/professional school, and graduate or professional degree. Rural status was dichotomized as yes/no. Logistic regression was used to identify factors that influence the use of combined nutritional CAM among participants. To better understand the factors associated with combined nutritional CAM use among older adults, we examined race, sex, age, and rural status, as these characteristics have been consistently identified in the literature as factors that influence CAM use for treatment, health promotion, and illness prevention (31–35). We also included CCI to assess the impact of chronic disease burden (36). Individuals managing multiple chronic conditions often experience persistent symptoms such as pain, fatigue, or inflammation, which may prompt them to seek nutritional CAM as a form of self-care. These covariates were selected to reflect established patterns in the literature and contribute to a deeper understanding of how demographic and disease burden characteristics influence the use of combined nutritional CAM among southern older adults.

## 3 Results

### 3.1 Participant characteristics

The descriptive statistics and participant characteristics are displayed in Table 1. Of the 419 participants, the majority identified as White (66%) and over half were women (58%). Forty-one percent resided in rural areas, with 42% of African American and 40% of White participants living in rural areas. Twenty-six percent of participants reported having a college degree. The mean age was 81.77 (SD 4.79), and the mean number of comorbidities, as measured by the CCI, was 2.68 (SD 1.59).

**Table 1:** Descriptive characteristics (*n* = 419).

Demographic characteristics	Mean (SD) or %
Age (years)	81.77 (4.79)
Charlson Comorbidity Index	2.68 (1.59)
Sex	
Female	57.6
Male	42.4
Race	
White	65.5
African American	34.5
Rural	
No	59.2
Yes	40.8
Education	
Less than High School	29.1
High School	29.8
Some College	20.0
College Graduate	8.1
Some Graduate/Professional School	2.9
Graduate or Professional Degree	10.0
Insurance	
Medicare	92.6
Medicaid	12.9
VA	7.9
Private	71.6

SD, Standard Deviation.

### 3.2 Chi-squared test of association between race, sex, and rural status

Chi-squared analysis revealed there was a small association between sex and use of combined nutritional CAM [X (2) (1) = 10.894, *p* < 0.001], Cramér’s V = 0.162, such that women were more likely to use combined dietary supplements, vitamins, minerals compared to men. Additionally, there was a small association between rural status and use of combined nutritional CAM [X^2^ (1) = 5.476, *p* = 0.019], Cramér’s V = 0.114; which suggests participants living in rural areas were less likely to use combined nutritional CAM. However, there were no significant associations between race and combined nutritional CAM. Although the association between rural status and the use of vitamins and minerals only was statistically significant [X^2^ (1) = 3.851, *p* = 0.050], the strength of the association was week (Cramér’s V = 0.096). However, there was a small association between sex [X^2^ (1) = 4.613, *p* = 0.032], Cramér’s V = 0.105; which suggests women were more likely to use vitamins and minerals only. There was no association between race and use of vitamins and minerals only. Lastly, there was a small association between sex and use of dietary supplements only [X^2^ (1) = 4.765, *p* = 0.029], Cramér’s V = 0.107. However, there were no significant associations between race and rural status for use of dietary supplements only.

### 3.3 Logistic regression

Table 2 shows the prevalence of nutritional CAM by groups, while Table 3 displays sociodemographic predictors of utilization by groups. Using a binary logistic regression model, our findings suggest a statistically significant association between sex (*p* < 0.001), age (*p* = 0.024), education (*p* < 0.001), and rural status (*p* = 0.008) in use of combined nutritional CAM (dietary supplements, vitamins, and minerals; *group 1*). Specifically, these findings suggest females were two times more likely to use combined nutritional CAM than men (OR = 2.00; CI: 1.31, 3.06). Furthermore, each additional increase in 1 year in age was associated with a 5% increase in the odds of using combined nutritional CAM (OR = 1.05; CI: 1.00, 1.09) and participants who lived in rural areas had 44% less odds of using combined nutritional CAM compared to those who did not live in rural areas (OR = 0.42; CI: 0.64, 0.14). Regarding education, participants who reported some college or above were more likely to use combined nutritional CAM (OR = 1.15; CI: 1.08, 1.23). However, race and CCI were not significant predictors for group 1.

**Table 2:** Prevalence of nutritional CAM by groups.

Type of nutritional CAM	Yes		No	
	*n*	%	*n*	%
Group 1 (Combined Nutritional CAM)	256	61.1	163	38.9
Group 2 (Vitamins and Minerals, only)	143	34.1	276	65.9
Group 3 (Dietary Supplements, only)	206	49.2	213	50.8

**Table 3:** Sociodemographic and comorbidity predictors by groups.

Covariates	Group 1 (*n* = 419)		Group 2 (*n* = 419)		Group 3 (*n* = 419)	
	Odds ratio	95% CI	Odds ratio	95% CI	Odds ratio	95% CI
Age at baseline	1.05	(1.017, 1.099)	1.087	(1.035, 1.141)	1.044	(1.000, 1.089)
Race	0.812	(0.514, 1.282)	0.688	(0.427, 1.110)	0.954	(0.605, 1.503)
Sex	2.003	(1.310, 3.064)	1.647	(1.060, 2.561)	1.501	(998, 2.256)
CCI	1.028	(0.908, 1.164)	0.966	(0.850, 1.097)	0.985	(0.873, 1.111)
Rural Status	0.557	(0.360, 0.861)	0.642	(0.409, 1.010)	0.942	(0.615, 1.445)
Education	1.157	(1.085, 1.233)	1.091	(1.022, 1.166)	1.118	(1.040, 1.202)
Insurance	1.655	(1.078, 2.540)	1.444	(0.912, 2.286)	1.352	(0.837, 2.184)

CCI, Charleson Comorbidity Index; CI, Confidence Interval. Group 1. Use of combined nutritional CAM (including, dietary supplements, vitamins, and minerals). Group 2. Use of vitamins and minerals, only. Group 3. Use of dietary supplements only.

For the use of vitamins and minerals only (*group 2*), our findings suggest a significant association between sex (*p* = 0.027), education (*p* = 0.009), and age (*p* < 0.001). This suggests, each increase in 1 year of age was associated with a decrease in the odds of using vitamins and minerals only (OR = 0.92; CI: 0.87, 0.97) and participants who reported some college or above were more likely to use vitamins and minerals only (OR = 1.09; CI: 1.02, 1.16). These findings also suggest that women were 1.6 times more likely to use vitamins and minerals only compared to men (OR = 1.64; CI: 1.06, 2.56). Race, rural status, and CCI were not statistically significant predictors for group 2.

Lastly, for the use of dietary supplements only (*group 3*), our findings suggest a significant association between age (*p* = 0.050) and education (*p* = 0.002). Specifically, participants who reported some college or above were more likely to use dietary supplements (OR = 1.1; CI: 1.04, 1.20) and each increase in 1 year of age was associated with a 4% increase in the odds of using dietary supplements, only (OR = 1.05; CI: 1.00, 1.09). Sex, race, CCI, and rural status were not significant predictors for group 3.

## 4 Discussion

Older adults often experience declining health with age (37, 38), and this decline is frequently compounded by the presence of multiple chronic conditions and nutritional deficiencies (6, 39). Nutritional CAM is widely marketed as a means to reduce the risk of non-communicable diseases, correct dietary imbalances, and alleviate minor aches and pains (40, 41). However, despite its popularity, the scientific evidence supporting the safety and effectiveness of many nutritional CAM modalities remains limited or inconclusive (11, 41). Given this gap, understanding sociodemographics and chronic disease burden factors associated with the use of combined nutritional CAM is crucial.

Our study found that women are more likely than men to use combined nutritional CAM, a finding that is consistent with previous research (6). However, the factors influencing the use of combined nutritional CAM varied by modality. For instance, regarding the use of dietary supplements only (group 3), the likelihood of use decreased with increasing age among women. Similarly, older age was associated with lower odds of using combined nutritional CAM (dietary supplements, vitamins, and minerals; group 1), a trend also observed among participants residing in rural areas compared to their urban counterparts. These findings diverge from the literature regarding trends in the use of dietary supplements only, highlighting the importance of examining combined nutritional CAM modalities. Although dietary supplement use is common among older adults, few studies have examined the concurrent use of dietary supplements, vitamins, and minerals in this population (42–44). To date, no studies have specifically focused on older adults, aged 75 and older, in the Southern United States. A notable gap given the region’s high burden of chronic disease in the aging population. This pattern may be influenced by the mean age of participants in the SOA II. Additionally, clinicians may advise against the use of dietary supplements, vitamins, and minerals for older adults due the potential harmful interactions with prescribed medications and the risk of exceeding recommended daily intake levels, depending on age, supplement type, and overall health status.

Furthermore, the literature suggests that CAM use varies across generational cohorts, reflecting differences in cultural influences and health beliefs. For example, Groden et al. suggest that individuals born in the late 1960s and early 1970s, often associated with the counterculture and social revolutions, tend to be more open to alternative health practices and may hold more progressive views on healthcare and service utilization (45). In contrast, those born in the 1930s, shaped by the Great Depression and more traditional values, may have distinct attitudes toward the use of combined nutritional CAM. These generational differences are shaped by distinct sociocultural events that influence values, trust in medical systems, and openness to non-conventional therapies (23).

Contrary to prior research, our findings revealed no statistically significant associations between race and the use of different nutritional CAM groups. This is particularly noteworthy given that previous studies have reported higher CAM use among White adults compared to African American adults, with varying patterns of use depending on CAM type (46, 47). The absence of racial differences in our study may reflect regional, cultural, or cohort-specific factors unique to our sample. Cultural determinants, such as beliefs, values, and norms, can influence how individuals perceive health and illness, and subsequently, their use of CAM. Regional factors, including access to healthcare resources and community norms, also affect CAM utilization. For example, adults in rural areas often report lower use of CAM, possibly due to limited availability or differing cultural perspectives (48). These intersecting factors may contribute to more uniform CAM usage patterns across racial groups, particularly among adults aged 75 and older. This underscores the need for further research to explore how race intersects with other sociodemographic and contextual variables in shaping combined nutritional CAM use. Lastly, although we initially hypothesized that higher CCI scores would be associated with increased use of nutritional CAM, this was not supported by the data. Across all three groups, CCI was not a statistically significant predictor of nutritional CAM use.

Findings from the current study have significant implications for the overall health of older adults, particularly women, and should inform both clinical practice and public health policy. As the popularity of self-initiated nutritional CAM continues to rise (49), there is a growing need for health professionals, including health educators, healthcare providers, and other allied health practitioners, to be equipped with evidence-based knowledge to guide patients safely. Certified allied health professionals, such as a Registered Dietitian Nutritionist (RDN), are uniquely positioned to provide science-based education on nutrition and malnutrition (30, 50). They can help older adults navigate the complex landscape of nutritional CAM, debunk misinformation, and assess the risks of combined CAM products. This is especially critical for older adults who often use nutritional CAM as part of broader self-management strategies for chronic conditions and general wellness (30). Clinicians are aware of potential interactions between nutritional CAM and prescription medications and should proactively engage patients in open, nonjudgmental discussions to ensure safe integration with conventional treatments. Moreover, integrating CAM education into curricula for nursing, nutrition, medicine, and pharmacy, and public health programs can prepare future professionals to support patient-centered care. This includes fostering shared decision-making, building trust, and promoting healthy aging practices through informed, evidence-based nutritional choices (51–54).

From a policy standpoint, there is a clear need for targeted educational initiatives in rural communities to provide accurate, accessible information about the benefits and risks of combined nutritional CAM. The NCCIH has emphasized a “whole person health” approach in its 2021–2025 strategic plan, which supports research and dissemination to understand the effectiveness and safety of nutritional CAM interventions to disseminate knowledge to healthcare professionals and the public (41). Additionally, improved data collection on CAM usage among older adults, with a particular focus on women, can inform more equitable and effective health interventions in rural areas. The NCCIH also supports initiatives like the NIH Pragmatic Trials Collaboratory and the HEAL Initiative, which aim to integrate nonpharmacologic approaches into mainstream care, particularly for pain and chronic disease management (55).

This study has several limitations. First, The SOA II is an epidemiological study that is representative of the general population of a Southern state in the United States. As such, the generalizability of the results may be limited, particularly to populations in other geographic regions or with different cultural and healthcare contexts. Although our sample was not fully representative of Alabama overall, considerable efforts were made to recruit African American participants, enhancing the diversity and relevance of the findings within the study region. A key methodological limitation involves the reliance on self-reported data, particularly regarding the use of nutritional CAM and changes in medication. Self-report measures are inherently subject to recall bias, social desirability bias, and misclassification. This concern is especially salient given the 36-month recall window used in the study. Older adults, who may experience varying degrees of cognitive decline or memory challenges, may have difficulty accurately recalling their use of nutritional CAM over an extended period. This could lead to underreporting or overreporting of nutritional CAM use, potentially skewing the observed associations. Additionally, we did not control for key health behavior variables such as physical activity, prescription drug use, or dietary habits. These behaviors are often closely associated with CAM use and may act as confounding or mediating variables in relation to utilization of combined nutritional CAM. Future studies should incorporate these factors to better understand the mechanisms at play. Despite these limitations, this study makes a novel contribution by examining the combined use of nutritional CAM therapies among Southern older adults and explores how sociodemographic and disease burden predict usage. To our knowledge, no prior study has investigated this combination of therapies in this population, highlighting the importance of continued research in this area.

## 5 Conclusion

This study explored the sociodemographic and chronic disease burden predictors of nutritional CAM use among older adults in the Southern United States. While prior research has documented widespread use of nutritional CAM in the general population, our findings reveal a notable decline in combined nutritional CAM use with advancing age. This trend persisted across key sociodemographic variables, suggesting that age itself may be a critical factor influencing combined nutritional CAM use in later life.

These findings carry important implications for health educators, clinicians, and public health professionals. As the older adult population continues to grow, understanding the nuanced patterns of combined nutritional CAM use, particularly in rural communities, will be essential for developing effective tailored interventions. Our results underscore the importance of designing educational interventions that support safe and informed nutritional CAM use among this population. Moreover, recognizing the diversity within nutritional CAM practices is crucial. Not all nutritional CAM are used for the same reasons, and future research should aim to capture this complexity with greater granularity. By doing so, we can better support older adults in making informed decisions that align with their health goals and values.

## Data Availability

The datasets presented in this article are not readily available because it contains personal identifiable information. Requests to access the datasets should be directed to the corresponding author, Kristen Allen-Watts.
